# Thoracic endograft–associated vertebral erosion

**DOI:** 10.1016/j.xjse.2026.100129

**Published:** 2026-04-30

**Authors:** Sotirios C. Kotoulas, Panteleimon Tsipas, Christophoros Kotoulas

**Affiliations:** a Joint Armed Forces Cardiac Surgery Department, 401 General Army Hospital of Athens, Athens, Greece; b Cardiology Department, Asklepeion General Hospital, Athens, Greece

**Figure:**
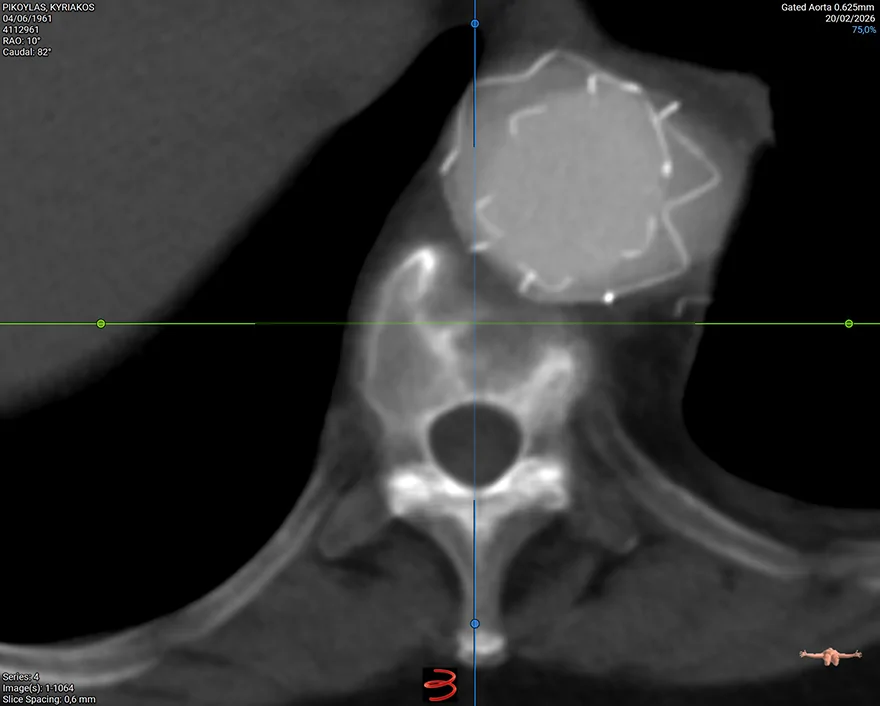
Axial CT showing TEVAR graft compressing the vertebral body with cortical erosion.

**Central Message**

Endotension after TEVAR may lead to progressive aneurysm sac expansion and vertebral erosion without endoleak, requiring multimodality imaging and multidisciplinary management.

A 65-year-old man with arterial hypertension and descending thoracic aortic aneurysm, without evidence of syphilitic, mycotic, or connective tissue etiology, was treated with thoracic endovascular aortic repair using 3 endografts. Approximately 3 years after the initial procedure, he presented with thoracic pain radiating to the back and left-lower extremity numbness. On admission, he was hemodynamically stable, without ischemic changes on electrocardiography and negative troponin levels. Transthoracic echocardiography demonstrated normal cardiac function, ascending aortic dilatation to 49 mm, and mild-to-moderate aortic regurgitation.

Coronary angiography was performed as part of preoperative cardiac evaluation in preparation for potential open surgical graft explantation and did not reveal obstructive coronary artery disease. Computed tomography aortography demonstrated enlargement of the aneurysm sac surrounding the thoracic endografts with T11 vertebral body erosion and loss of the intervening fat plane (Figure 1, Videos 1 and 2). There was no history of renal insufficiency, osteoporosis, or other metabolic bone disorders, whereas serum calcium and phosphorus values were within normal ranges.

**Figure 1:**
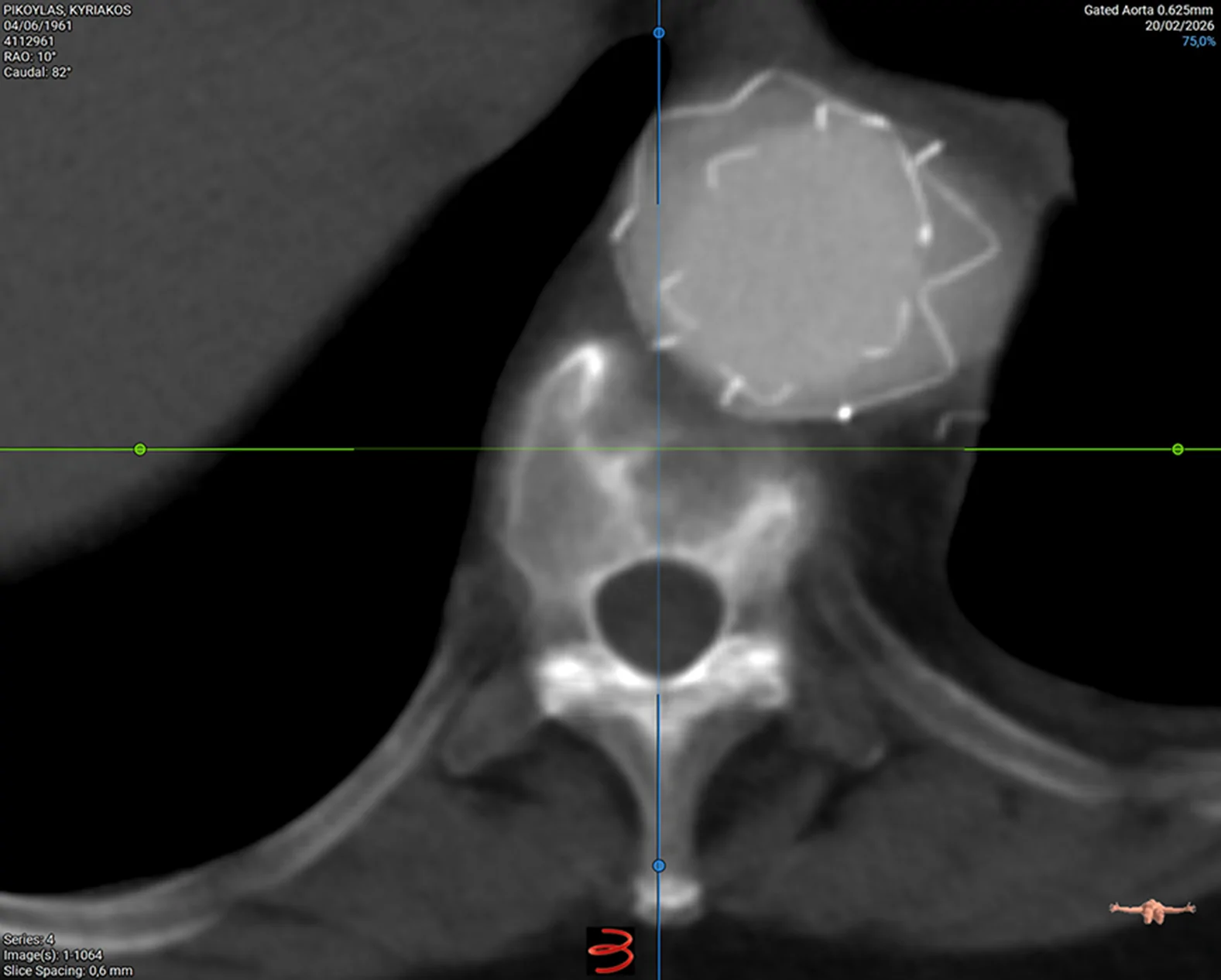
Axial computed tomography scan shows a thoracic endovascular aortic repair graft compressing the vertebral body with cortical erosion.

Multiplanar reconstruction confirmed direct contact between the aneurysm sac and vertebral cortex. Digital subtraction angiography demonstrated no endograft endoleak. Magnetic resonance imaging confirmed cortical destruction of T11 without epidural abscess or spinal cord compression (Figure 2).

**Figure 2:**
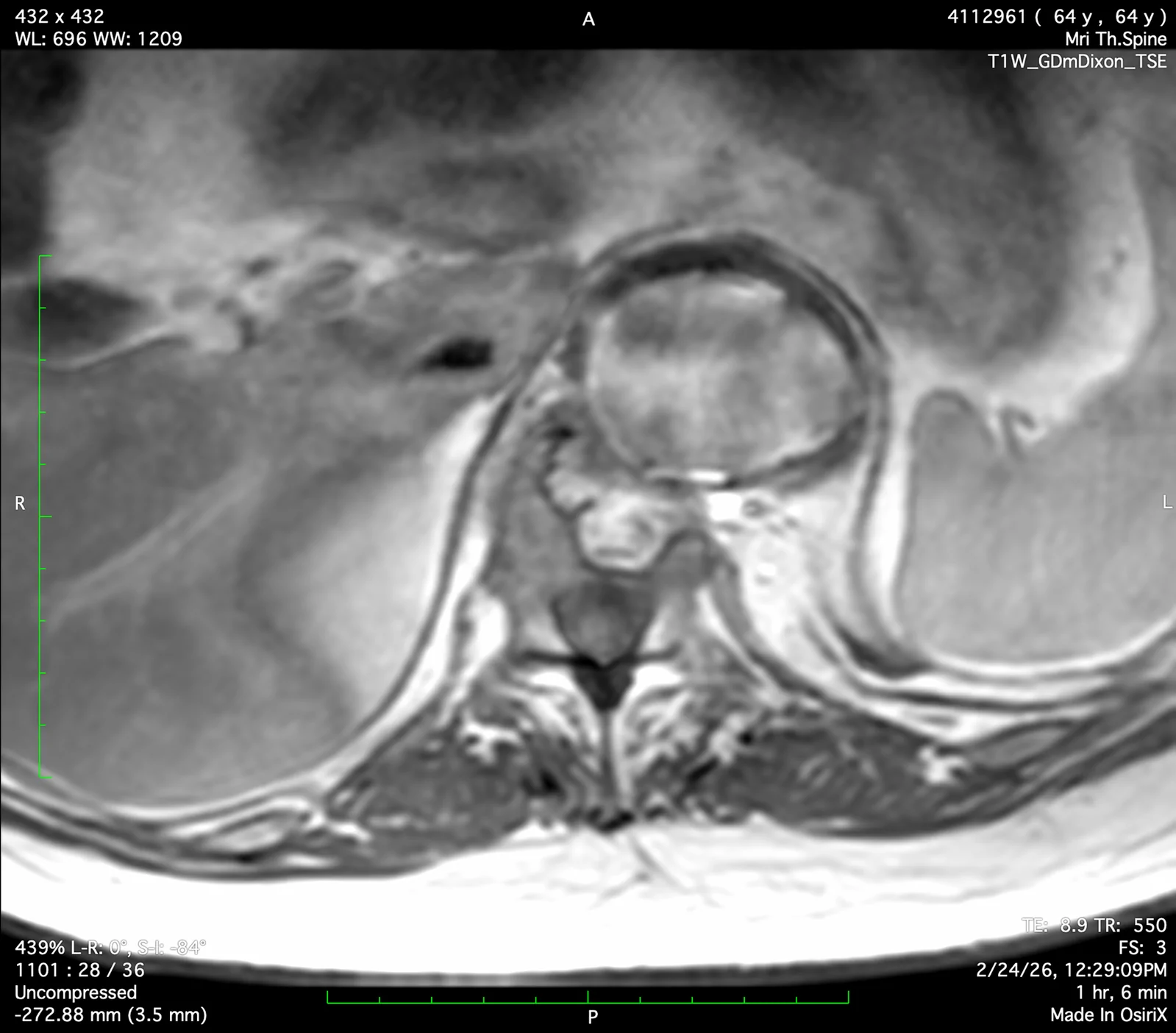
T1-weighted magnetic resonance imaging scan shows vertebral body erosion adjacent to the thoracic endovascular aortic repair aneurysm sac.

Multidisciplinary evaluation was performed. In the absence of endoleak and given progressive structural vertebral destruction, high operative risk of graft explantation, and the patient's preference, spinal stabilization was recommended to prevent mechanical instability and neurologic compromise.

Progressive aneurysm sac enlargement after thoracic endovascular aortic repair without detectable endoleak is described as endotension.^1^ Progressive sac expansion may result in structural complications, including vertebral erosion.^2^ The proposed mechanism of vertebral erosion is the rhythmic pulsation of the aorta against the adjacent vertebral body. In each cardiac cycle, the pulsatile force exerts repetitive mechanical stress on the vertebral cortex, leading to progressive pressure erosion.

## Conflict of Interest Statement

The authors reported no conflicts of interest.

The *Journal* policy requires editors and reviewers to disclose conflicts of interest and to decline handling or reviewing manuscripts for which they may have a conflict of interest. The editors and reviewers of this article have no conflicts of interest.

## Supplementary Data

Video 1 Three-dimensional reconstruction of the spine revealing the erosion. Video available at: https://www.jtcvs.org/article/S2950-6050(26)00034-3/fulltext.

Video 2 Three-dimensional reconstruction of the spine and the aorta. Video available at: https://www.jtcvs.org/article/S2950-6050(26)00034-3/fulltext.
